# The evolution of the class A scavenger receptors

**DOI:** 10.1186/1471-2148-12-227

**Published:** 2012-11-27

**Authors:** Fiona J Whelan, Conor J Meehan, G Brian Golding, Brendan J McConkey, Dawn M E Bowdish

**Affiliations:** 1 Department of Pathology and Molecular Medicine, McMaster University, 1200 Main Street West, Hamilton, L8N 3Z5, Ontario, Canada; 2 Faculty of Biochemistry and Molecular Biology, Dalhousie University, 5080 College Street, Halifax, Nova Scotia, Canada, B3H 4R2; 3 Department of Biology, McMaster University, 1280 Main Street West, Hamilton, Ontario L8S 4L8, Canada; 4 Department of Biology, University of Waterloo, 200 University Avenue West, Waterloo, N2L 3G1, Ontario, Canada

**Keywords:** Class A scavenger receptor, Innate immunity, Scavenger receptor, Pattern recognition receptor, Scavenger receptor cysteine rich domain, Comparative evolution

## Abstract

**Background:**

The class A scavenger receptors are a subclass of a diverse family of proteins defined based on their ability to bind modified lipoproteins. The 5 members of this family are strikingly variable in their protein structure and function, raising the question as to whether it is appropriate to group them as a family based on their ligand binding abilities.

**Results:**

To investigate these relationships, we defined the domain architecture of each of the 5 members followed by collecting and annotating class A scavenger receptor mRNA and amino acid sequences from publicly available databases. Phylogenetic analyses, sequence alignments, and permutation tests revealed a common evolutionary ancestry of these proteins, indicating that they form a protein family. We postulate that 4 distinct gene duplication events and subsequent domain fusions, internal repeats, and deletions are responsible for the diverse protein structures and functions of this family. Despite variation in domain structure, there are highly conserved regions across all 5 members, indicating the possibility that these regions may represent key conserved functional motifs.

**Conclusions:**

We have shown with significant evidence that the 5 members of the class A scavenger receptors form a protein family. We have indicated that these receptors have a common origin which may provide insight into future functional work with these proteins.

## Background

**Figure 1:**
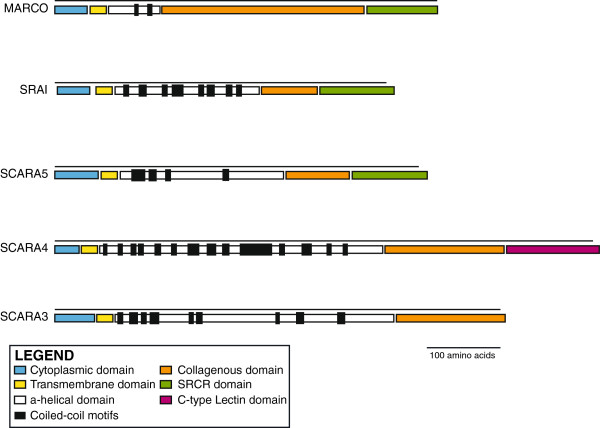
**The protein domain architecture of the class A scavenger receptors.** Structures are scaled based on the length of each domain. The cytoplasmic and transmembrane domains were determined using TMHMM software; *α*-helical domains were determined using the JUFO Server and PSIPRED. The boundaries of the collagenous, SRCR, and C-type lectin domains were determined using NCBI’s CDD. Heptad coiled-coil motifs of the *α*-helical domains were identified based on the definition put forth in [3,4]. Domain boundaries are supported by Additional file 1: Table S2.

The scavenger receptors (SRs) are a structurally diverse group of pattern recognition receptors (PRRs) which were originally defined based on their ability to bind and subsequently internalize acetylated low-density lipoprotein (acLDL). These receptors have since been shown to have the ability to bind some (but not all) polyanions [1-4] including ligands on modified host proteins and apoptotic cells [5]. Since their initial discovery in 1979 [1], a variety of proteins have been included in the SR family based on their ligand binding capabilities and/or similarities in their secondary structures, resulting in a diverse family of seemingly unrelated proteins [6,7]. Consequently, in 1997 Krieger suggested that the SRs be divided into 8 distinct classes, termed A through H, on the basis of protein sequence comparisons and domain architecture [6]. The class A scavenger receptors (cA-SRs) consist of 2 original members, namely Scavenger Receptor class A I (SRAI) and MAcrophage Receptor with COllagenous domain (MARCO) [6]. Three additional members have been subsequently added: Scavenger Receptor class A, member 3 (SCARA3)/CSR (Cellular Stress Response), SCARA4/SRCL (Scavenger Receptor with C-type lectin domain), and SCARA5 [8-10]. The cA-SRs are type II glycoproteins consisting of an intracellular N-terminal domain and extracellular C-terminus [11]. All 5 members form homotrimers that are thought to be stabilized via *α*-helical coiled-coil motifs in addition to their collagenous regions [12-14]. In general, the cA-SRs have similar domain structures with some obvious exceptions at the C-terminal end (Figure 1; Additional file 1: Table S2). These proteins vary considerably in the length of their collagenous domains, ranging from approximately 75 residues in SCARA5 to 250 amino acids in MARCO [10,15]. Importantly, the C terminus domain varies between the members of this group. SRAI, MARCO, and SCARA5 possess a terminal Scavenger Receptor Cysteine Rich (SRCR) domain [5], whereas SCARA3 terminates at the collagenous domain [8], and SCARA4 possesses a C-type lectin domain [9].

Alongside the C-type lectin domain of the collectins [16] and the leucine-rich repeat of the Toll-like receptors (TLRs) [17], the SRCR domain is one of the most ancient pattern recognition domains associated with innate immunity [18]. This domain possesses 6 highly conserved cysteine residues resulting in a distinctive pattern of disulfide bonding [18].

The SRCR domain is not restricted to the cA-SRs and is instead part of many other proteins across deuterosomes. These other SRCR-containing proteins have been implicated in a wide variety of functions, including pathogen recognition, endocytosis, and immune response homeostasis (reviewed in [18]); however, the role of the SRCR domain in the cA-SRs remains unclear. Studies of MARCO and SRAI implicate a region of the SRCR domains as a potential ligand binding motif [19,20]. In contrast, other mutagenic studies have shown that the collagenous region is sufficient for the binding of acLDL [13,21]. Whether this discrepancy is due to the particular ligands examined and/or multiple binding motifs is unknown.

While SRAI and MARCO are primarily expressed on macrophages [15,22], SCARA3, SCARA4, and SCARA5 are expressed on a variety of other cell types, including epithelial cells [10], and cells of the placenta, lungs, heart, and small intestine [9]. SRAI is primarily implicated in homeostatic functions such as the uptake of modified lipids and proteins, in addition to having a role in pathogen clearance [12,14]. In contrast, MARCO has been primarily implicated in host defense via the direct recognition and subsequent endocytosis of pathogens and the modulation of cytokine production [23,24]. Both SCARA4 and SCARA5 have been documented *in vitro* to bind bacteria [9,10], although this ability has not been established *in vivo*. Conversely, SCARA3 has been associated with the protection of cells from reactive oxygen species during oxidative stress [8]. This combination of diverse patterns of expression and function raise questions regarding whether these proteins are related to one another.

The scavenger receptors were originally grouped based on their ability to bind acLDL as a ligand, even if this binding ability can have very low affinity [25]. This broad and imprecise definition, which ignores the diversity of their biological functions and expression patterns, raises the question of whether these proteins share any evolutionary relatedness. In this study, we present multiple evolutionary and phylogenetic analyses of the cA-SRs by mining publicly available genomes for these receptors. We discovered that there is no evidence of cA-SRs in non-vertebrate species, suggesting that the domain architecture of this protein family is unique to that of vertebrates. To our knowledge, these are the first examples of thorough evolutionary analyses of this family. Our results confirm that an evolutionary relationship exists between all 5 members of the cA-SRs. We postulate that 4 unique gene duplication events, followed by domain fusions, internal repeats, and deletions, shaped the current architecture of this family to include some diversity in structure and function.

## Results and discussion

### The cA-SRs share similar domain architectures

Sixteen SRAI, 21 MARCO, 21 SCARA3, 25 SCARA4, and 22 SCARA5 full-length mRNA and protein sequences were identified and analyzed in this study (Additional file 2: Table S1). An exhaustive bioinformatic search was undertaken in order to identify these receptors, including searches of all SRCR-containing proteins for transmembrane, *α*-helical, and collagenous domains using various bioinformatic tools. These extensive methods were used in order to best identify any ancient homologs, pseudogenes, or related proteins that had undergone various domain swap or fusion events. Many of the cA-SRs examined have not been previously annotated and therefore represent novel cA-SR sequences. Previous analyses of the domain structures of the cA-SRs have been inconsistent; therefore, we re-examined these predictions using current bioinformatic tools. Although the domain architectures were resolved for each scavenger receptor sequence, those from the *Homo sapiens* genome were used as representatives to visualize the relative lengths and composition of these domains in Figure 1 and explained in detail in Additional file 1: Table S2. Cytoplasmic and transmembrane domains were established using the TMHMM software [26] and were determined to be approximately 30-55 and 20 amino acids long, respectively, in each receptor.

Previous work indicated the region between the transmembrane and collagenous domains to be a combination of a spacer and *α*-coiled-coil region dependent on the receptor in question (reviewed in [5]). Our analyses using the JUFO Server ( http://www.jens-meiler.de/jufo.html) and PSIPRED [27] indicated that this region is primarily *α*-helical in all 5 receptors and includes multiple coiled-coil motifs (Figure 1, black boxes). The coiled-coil motifs are based on heptad motifs of the form HxxHcccH [3,4], where hydrophobic residues (H) appear at the first and fourth positions of a seven amino acid sequence, with positions five to seven tending to be charged (c). Variations on this 3-4 separation pattern of hydrophobic residues include 4-4, 3-3, and 3-1 repeats [4]. These motifs have been shown to be necessary for oligomerization in other proteins [3] and thus are likely to contribute to the trimerization of the cA-SRs.

The boundary between this *α*-helical domain and the collagenous region was determined using the characteristic Gly-Xxx-Yyy repeat (reviewed in [5]), which appears over the full-length of the collagenous domain. The C-terminal domains have been previously annotated in NCBI and were confirmed using NCBI’s CDD. The resulting domain architecture shows strong similarities across the cA-SR protein family.

### Classification of known and novel cA-SRs

Bayesian and maximum likelihood phylogenies were constructed for each of the 5 protein family members using full-length protein sequences of the known and novel cA-SRs gathered from available genomes present in the NCBI and Ensembl databases. Novel cA-SRs were identified based on domain structure, synteny analyses, and pairwise sequence identity scores as compared to known cA-SRs. Phylogenies of these sequences were created to examine and confirm the within group relatedness of these proteins across vertebrate species.

The molecular phylogeny of full-length MARCO protein sequences (Additional file 3: Figure S1a) details the conservation of MARCO across mammalian and avian species. A partial transcript of a MARCO-like gene covering the SRCR and a piece of the collagenous domain was found in the *Xenopus tropicalis* genome, indicating that a functional MARCO gene might also be present in amphibians (Additional file 2: Table S1). However, the sequence was excluded from further analyses since the full-length protein sequence spans multiple contigs and could not be reliably constructed. Similarly, SRAI is present in mammalian and amphibian genomes (Additional file 3: Figure S1b), yet there appears to be a secondary loss of SRAI in avian species since it is absent from the *Gallus gallus* and *Meleagris gallopavo* genomes. SCARA5 appears to be the most abundant of the SRCR-containing cA-SRs, as the gene is conserved in mammals, birds, amphibians, reptiles, and fish (Additional file 3: Figure S1c).

Both of the non-SRCR-containing cA-SRs, SCARA3 and SCARA4, are also present in mammalian, avian, amphibian, reptilian, and fish genomes. Of the 2 proteins, SCARA3 (Additional file 3: Figure S1d) was found in Ostariophysian and Salmonidae fish species, while SCARA4 (Additional file 3: Figure S1e) is present in these genomes as well as the bony Acanthopterygii fishes.

### MARCO, SRAI, and SCARA5 share a highly conserved SRCR domain

**Figure 2:**
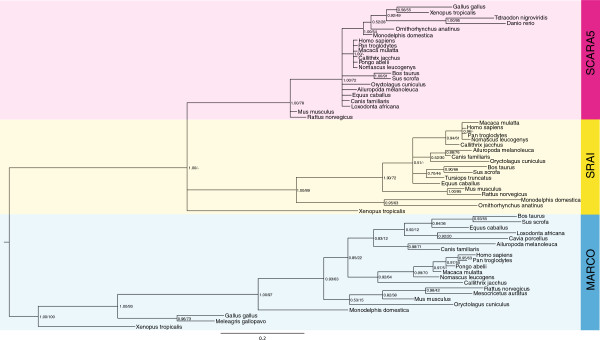
**The SRCR domain is highly conserved across the SRCR-containing class A scavenger receptor protein sequences.** A phylogeny built using both Bayesian and maximum likelihood methods demonstrates the relatedness of the protein SRCR domain sequences across MARCO (blue), SRAI (yellow), and SCARA5 (red). Node labels indicate both posterior probabilities generated from the Bayesian analysis and bootstrap values of the maximum likelihood tree [ML/BY]. Phylogeny is midpoint rooted; scale bar indicates the number of substitutions per site.

Three of the cA-SRs (MARCO, SRAI, and SCARA5) possess an evolutionarily conserved SRCR domain. The SRCR domain is present in many proteins and is highly conserved across various deuterosome species [18]. Phylogenetic analysis of the SRCR domains from these 3 cA-SRs were conducted in order to determine the evolutionary relations between them. By both Bayesian and maximum likelihood methods, the SRCR domains of each receptor cluster together, with those domains from SRAI grouping closer to those of SCARA5 when compared to MARCO (Figure 2), indicating that the SRCR domains of SRAI and SCARA5 are more similar to each other than to those of MARCO and are likely to have diverged from a more recent common ancestor.

### The non-SRCR containing cA-SRs - SCARA3 and SCARA4 - are evolutionarily related to each other

**Figure 3:**
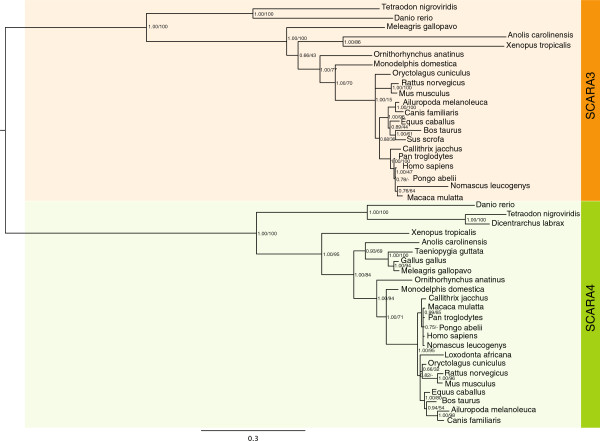
**Phylogenetic analysis of the domains shared by SCARA3 and SCARA4 protein sequences.** A phylogeny built using both Bayesian and maximum likelihood methods demonstrates the clustering of SCARA3 (orange) and SCARA4 (green) proteins across vertebrate species. Node labels indicate both posterior probabilities generated from the Bayesian analysis and bootstrap values of the maximum likelihood tree [ML/BY]. Phylogeny is midpoint rooted; scale bar indicates the number of substitutions per site.

Of the 5 cA-SRs two, SCARA3 and SCARA4, do not possess the conserved SRCR domain at their C-terminus. Instead, SCARA4 has a C-type lectin domain and SCARA3 terminates after its collagenous region. Permutation tests of *Homo sapiens* SCARA3 and SCARA4 confirmed that their full-length amino acid sequences are statistically similar to each other (Table 1). Further phylogenetic analyses of the domains shared between these 2 cA-SRs determined the clustering of SCARA3 and SCARA4 sequences across vertebrate species (Figure 3).

**Table 1:** **Percent identity and permutation test scores between the full-length *Homo sapiens *cA-SR amino acid sequences**

	**SRAI**	**MARCO**	**SCARA3**	**SCARA4**	**SCARA5**
**SRAI**		25.0%	13.5%	14.7%	32.3%
**MARCO**	5.37e-18		12.9%	21.8%	25.0%
**SCARA3**	4.26e-13	1.04e-12		26.6%	18.5%
**SCARA4**	3.66e-17	1.34e-16	7.23e-54		15.7%
**SCARA5**	4.88e-52	1.06e-26	8.70e-15	5.34e-25	

The percent identity (top right) between each cA-SR was calculated using EBI’s EMBOSS Needle global alignment algorithm to quantify the amount of sequence similarity shared amongst these receptors. Additionally, permutation tests were measured using the PRSS algorithm part of the FASTA package. The probabilities displayed here (bottom left) are the probability that these receptors share sequence similarity with each other by chance.

### A common ancestry is shared between all 5 members of the cA-SRs

**Figure 4:**
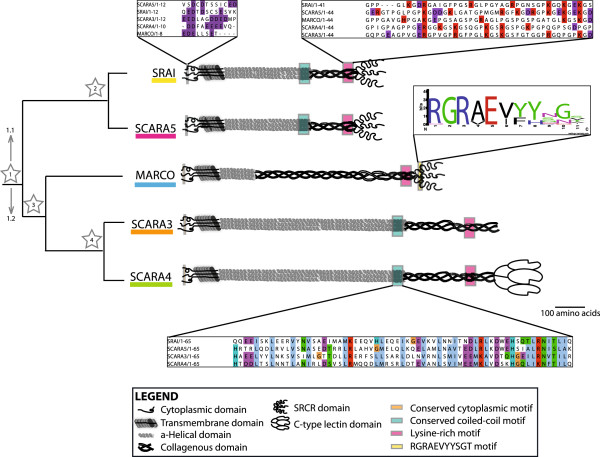
**A summary of the common motifs in the class A scavenger receptor protein sequences.** Conserved motifs present in the protein sequences of these receptors are indicated with coloured boxes at their approximate position within the protein with shout out boxes used to show the level of conservation across the aligned *Homo sapiens* sequences.

Permutation tests performed using the PRSS software established that each *Homo sapiens* cA-SR amino acid sequence is statistically similar to each other, establishing a strong evolutionary relationship connecting all members of this family (Table 1). Additional analyses of similarities across the cA-SR *Homo sapiens* amino acid sequences confirmed significant sequence similarity amongst these proteins. Analyses identified 4 conserved motifs including a cluster of negatively charged amino acids in the cytoplasmic domain of the 5 cA-SRs (Figure 4, orange boxes). Furthermore, in addition to the plethora of coiled-coil heptad motifs, a conserved motif in the *α*-helical domains of each receptor, excluding MARCO [13], was established (Figure 4, teal boxes). A previously predicted ligand-binding motif of MARCO [19] was not found in the SRCR domains of SRAI and SCARA5 (Figure 4, yellow box); however, the lysine-rich region in the collagenous domain of SRAI hypothesized to be necessary for ligand binding was found in all other cA-SRs (Figure 4, pink boxes). These similarities in domain structure and conserved motifs support a common evolutionary relationship between all 5 cA-SRs.

Further support for a common evolutionary origin is seen in shared exon features in cA-SR members (Additional file 4: Table S3). Each of the 5 cA-SR types contains similar overall architecture and exon order, including (in order) a cytoplasmic region, transmembrane domain, *α*-helical region, and collagenous region. The single exon containing a portion of the cytoplasmic region plus the transmembrane domain is conserved across all 5 cA-SR types. Exons corresponding the *α*-helical and collagenous regions are also present in all types, and have undergone expansion and/or contraction in various family members. Notably, the collagenous region of MARCO has expanded considerably and contains numerous additional exons. The *α*-helical region has also undergone expansion and contraction, with expansion likely occurring within an existing exon in the SCARA3/SCARA4 branch and reduction occurring within MARCO.

### The evolutionary history of the cA-SRs

**Figure 5:**
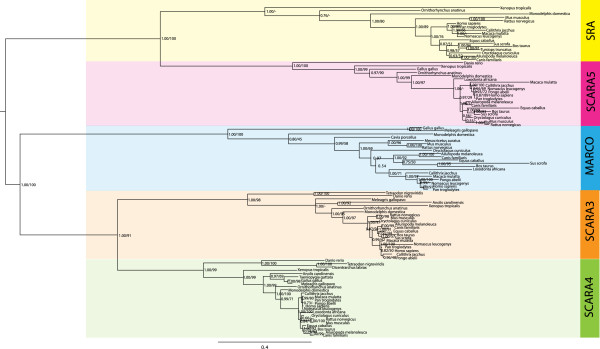
**Phylogeny of all the common domains shared by the class A scavenger receptor protein sequences.** Bayesian and maximum likelihood phylogenetic analyses of SRAI (yellow), SCARA5 (red), MARCO (blue), SCARA3 (orange), and SCARA4 (green) protein sequences show a possible evolutionary history of this protein family. Node labels indicate both posterior probabilities generated from the Bayesian analysis and bootstrap values of the maximum likelihood tree [ML/BY]. Phylogeny is midpoint rooted; scale bar indicates the number of substitutions per site.

In order to specify the exact relationships amongst the members of the cA-SR gene family, a phylogeny was established using the 4 domains shared across these receptors (Figure 5). This phylogeny suggested a strong relationship amongst SCARA3 and SCARA4 in addition to between SRAI and SCARA5, and that MARCO amino acid sequences cluster between the non-SRCR containing receptors and SRAI and SCARA5. Pairwise identity scores were calculated between each full-length *Homo sapiens* cA-SR protein sequences (Table 1) which identify a higher level of similarity between MARCO and the other SRCR-containing receptors when compared to between MARCO and the non-SRCR-containing proteins.

## Discussion

Since their discovery in 1979, scavenger receptors have been defined by their ability to ‘scavenge’ modified LDL from their environment for internalization and subsequent degradation [1]. As more proteins were discovered that fit this definition, the SRs came to represent a polyphyletic group of receptors with varying domain architectures and protein structures that appear to have arose independently (for example, although CD36, a class B SR, also binds modified lipids, permutation tests show that it is unrelated to SRAI (data not shown)). This prompted the introduction of subclasses to group structurally similar proteins [6]. However, even within the class A subclass there is considerable variability. Functionally, for example, MARCO can bind acLDL [23], SRAI can bind both oxLDL and acLDL [28], and SCARA5 can bind neither [10]. Structurally, the cA-SRs differ at their C-terminal region and in the lengths of their other domains (Figure 1). There is very little justification for grouping the cA-SRs together based on the original definition of ligand binding unless there is an evolutionary relationship amongst the members.

To investigate the evolutionary connection within the cA-SRs, we first needed to definitively characterize the domain architecture of these proteins. Domain boundaries had been previously defined for the individual members of the cA-SRs, but usually in comparison to SRAI and were not based on current tools. Our findings (Figure 1, Additional file 1: Table S2) suggest that there are 4 domains - cytoplasmic, transmembrane, *α*-helical, and collagenous - shared by all members of the cA-SRs. Conserved motifs in these domains common across the cA-SRs suggest not only a common origin of these proteins, but also that they may share significant functionality with each other (Figure 4). While the lengths and consistency of the cytoplasmic and transmembrane domains remain mostly fixed, the *α*-helical and collagenous domains vary in length across the receptors in a manner consistent with the possibility of repeats brought about by recombination or duplication events [29]. In contrast, the fifth terminal domain differs or is absent in the cA-SRs. While SRAI, MARCO, and SCARA5 share a SRCR domain at their terminus, SCARA4 possesses a C-type lectin domain and SCARA3 terminates at its collagenous region. The SRCR and C-type lectin domains are both able to recognize pathogens [18,30], suggesting that the radiation in this region may be due to a domain swapping event that may have allowed for the diversification of host pathogen recognition [31].

Data mining was used to identify known and novel cA-SRs in publicly available databases. Conservation of these proteins across vertebrate species was examined via phylogenetics. No cA-SRs were identified in available non-vertebrate genomes, implying that although the individual domains that make up these receptors - specifically the SRCR and C-type lectin domains - are ancient, the modern cA-SR domain architecture likely arose after the divergence of vertebrates from other species. Using these sequences, the relationships between the 5 members of the cA-SRs were analyzed.

To determine a shared evolutionary ancestry amongst all 5 members of the cA-SRs, permutation tests were performed using the representative *Homo sapiens* protein sequences, which revealed significant sequence similarity between all of these proteins (Table 1). Additionally, notable motifs shared amongst all or most receptors were identified (Figure 4), lending definitive reason for these proteins to be classified as a protein family.

Phylogenetic analyses allowed us to hypothesize regarding the evolutionary history of this protein family. First, analyses presented in Figures 2, 4, and 5 indicate that SRAI and SCARA5 are most closely related to each other than to the other cA-SRs. This finding is further supported in the fact that the highest amount of sequence similarity is shared between SRAI and SCARA5 (Table 1). This is unsurprising given what is known biologically about these 2 proteins. Although little research has been completed on SCARA5, it is known that both it and SRAI bind Gram-positive and -negative bacteria [10,28,32] and are both hypothesized to be involved in host defense [10,33]. Second, SCARA3 and SCARA4 were also identified as closely related proteins. Not only are their domain lengths similar (Figure 1), but these proteins are also presented as an independent cluster in the phylogenetic analysis of all cA-SRs (Figure 5). Although they are not well studied, from what we know these 2 proteins do not share much functionality. From what little is known regarding SCARA4, this receptor appears to function in a similar fashion to the SRCR-containing cA-SRs by binding Gram-positive and -negative bacteria and being expressed on cells involved in host defense [9,34]. In contrast, SCARA3 is expressed on fibroblasts and has been proposed to protect against reactive oxygen species by binding and internalizing oxidative molecules [8]. However, the lengths and general composition of SCARA3 and SCARA4 proteins are very similar as indicated by a shared percent identity of 26.6% across the full-lengths of their proteins (Table 1). Perhaps the differences in their biological functions are restricted to the presence of a C-terminal C-type lectin domain in SCARA4 and the potentially lost terminal domain in SCARA3.

Lastly, the positioning of MARCO is intermediate between the SRAI/SCARA5 and SCARA3/SCARA4 clusters. The phylogenetic evidence presented in Figure 5 suggests that this protein sequence is most similar to SCARA3/SCARA4 with high posterior probabilities and bootstrap support. However, percent identity measures (Table 1) as well as functional evidence suggests that it is most similar to the other SRCR-containing receptors. For example, research conducted by Arredouani *et al.* demonstrates that both SRAI and MARCO are essential for clearance of bacteria and inert particles from the lungs [24,35], indicating that even though MARCO is more evolutionarily related to SCARA3 and SCARA4, it is more functionally related to the SRCR-containing receptors. Further analysis of the exon gene structures of the cA-SRs or further functional analyses of all 5 members may help resolve this inconsistency.

This data supports the hypothesis of a single ancestral cA-SR from which duplication events occurred allowing for the diversification of this group. We propose that 4 independent gene duplication events occurred allowing for the presence of 5 cA-SRs in vertebrate species. This common ancestor likely included most of the common features of the cA-SRs including the transmembrane, *α*-helical, and collagenous domains, and may have also contained the SRCR domain shared by 3 of the 5 cA-SRs. This ancestral cA-SR may have duplicated (Figure 4, Event 1) into 2 distinct proteins (labelled 1.1 and 1.2) which would have contained the domain structure typical of this group (i.e. cytoplasmic, transmembrane, collagenous, and C-terminal domains). A second duplication event of putative proto-gene 1.1 (Figure 4, Event 2) would have resulted in the genes that differentiated into SRAI and SCARA5. The putative 1.2 gene would have contained an SRCR coding domain, and possibly an extended collagenous region (as compared to 1.1). This SRCR encoding region would likely have been lost in the predecessor of SCARA3 and SCARA4 upon a third duplication event, which would have resulted in the ancestral gene encoding MARCO (Figure 4, Event 3). The SRCR domain may have been replicated by a C-type lectin domain in the predecessor of SCARA3 and SCARA4 and later lost in SCARA3 when a fourth duplication event resulted in the divergence from SCARA3 and SCARA4 (Figure 4, Event 4), or may have simply replaced the C-type lectin of SCARA4.

## Conclusions

Despite the broad, general definition that brought the 5 members of the cA-SRs into the same subclassification of proteins capable of recognizing modified lipoproteins, we have shown significant evidence here that these 5 proteins are indeed a protein family. There is considerable evidence of a common origin for these proteins, which may in turn provide insight when performing functional studies on members of this family.

## Methods

### Mining and annotation of class A scavenger receptor mRNA and amino acid sequences

Deuterosome genomes from NCBI’s GenBank ( http://www.ncbi.nlm.nih.gov/genbank/) and EBI’s Ensembl ( http://www.ensembl.org) databases were analyzed for novel cA-SR amino acid sequences. First, the protein sequences of known cA-SRs were used as queries to the Basic Local Alignment Search Tool (BLAST) [36] with an initial E-value cut-off of 10^−30^ in order to identify orthologs. From this list of proteins, cA-SRs were identified as consisting of a C-terminal SRCR domain in the case of MARCO, SRAI, and SCARA5, or a C-type lectin domain in the case of SCARA4, connected to a collagenous region, consisting of at least 70 amino acids in length. Additionally, significant sequence similarity between the identified ortholog and known cA-SRs had to be shared as defined by a percent identity score greater than 20% using the Needleman–Wunsch algorithm. In the case of SCARA3, proteins were annotated based solely on full-length sequence similarity to known SCARA3 sequences. Further, Position-Specific Iterated BLAST (PSI-BLAST) [37] and the BLAST-like alignment tool (BLAT) [38] tools were used with default values (PSI-BLAST threshold of 0.005) to ensure all novel cA-SRs were discovered from publicly available genome information. Additional gene synteny analyses were conducted with the aid of the UCSC Genome Browser [39] when only partial sequences were available. When appropriate, publicly available predicted transcript data were manually edited to reflect known cA-SR exon structure. In the case where only partial sequences were available, the sequences were omitted from further analyses. Multiple sequence alignments of the cA-SR mRNA and amino acid sequences were generated using MUlitple Sequence Comparison by log-exception (MUSCLE) [40] and viewed using JalView 6.7.1 [41]. Known and newly annotated cA-SR sequences are presented in Additional file 2: Table S1.

### Domain characterization and similarity measures

In order to determine the domain architecture of each cA-SR, the boundaries of each domain were calculated using bioinformatic software. The cytoplasmic and transmembrane domains were determined with TMHMM2.0 [26]. The *α*-helical regions were identified with the JUFO Server ( http://www.jens-meiler.de/jufo.html) and PSIPRED [27]. The collagenous, SRCR, and C-type lectin domain boundaries were determined via NCBI’s Conserved Domain Database (CDD) [42]. Additionally, permutation tests to compare each of the *Homo sapiens* cA-SR amino acid sequences were generated using PRSS with 1000 iterations [43,44]. Percent identity measures calculated for the same sequences were based on pairwise distance scores calculated using EBI’s EMBOSS Needle global alignment algorithm using default settings [45].

### Construction of phylogenetic trees

Molecular phylogenies of the cA-SR mRNA and amino acid sequences were created using both maximum likelihood and Bayesian probabilistic methods of evolution. These methods were implemented using the RAxML-VI-HPC v7.2.8 [46] and MrBayes 3.1.2 [47,48] software packages, respectively. The appropriate substitution models for each phylogeny were determined by jModelTest [49] and ProtTest [50]. The MARCO mRNA data were estimated to fit most appropriately with the Generalized Time-Reversible (GTR) model including both invariable sites (I) and a discrete gamma (G) distribution. All other mRNA data were estimated to be best represented by the GTR + G model. To create the phylogenies for gene trees based on full-length mRNA sequences, MrBayes analyses were run for 3 million generations; for all other comparisons, MrBayes was run for 10 million generations. All Bayesian phylogenies were sampled every 1000 generations and a 25% burn-in period was used. Convergence was confirmed by use of the AWTY [51] software package and variation in likelihood values were visualized using Tracer v1.5 [52]. Maximum likelihood phylogenies were also created using the appropriate substitution models and were subject to 100 bootstrap replicates. All trees were mid-point rooted using FigTree v1.3.1 [53].

## Abbreviations

SCARA: Scavenger Receptor class A; SRCR: Scavenger Receptor Cysteine Rich; acLDL: acetylated low density lipoprotein; oxLDL: oxidized low density lipoprotein; MARCO: Macrophage Receptor with Collagenous domain; SRAI: Scavenger Receptor class A I; CDD: Conserved Domain Database.

## Competing interests

The authors have no competing interests to declare.

## Authors’ contributions

FJW, DMEB, and BJM conceived and designed experiments. FJW carried out experiments, including all database mining, phylogenetic studies, permutation tests, and sequence alignment analyses. FJW and CJM analyzed experiments. CJM and GBG provided advice on experimental design and analysis. FJW and DMEB drafted the manuscript. All authors read, edited, and approved the final manuscript.

## Supplementary Material

Additional file 1 **Table S2.** Domain boundaries of the representative class A scavenger receptors in *Homo sapiens*. Probabilities for the cytoplasmic and transmembrane domains were determined using the TMHMM software tool. The *α*-helical domain with coiled-coil motifs was determined using the JUFO Server and PSIPred [JUFO;PSIPRED]. The collagenous, SRCR, and C-type lectin domains were determined using NCBI’s CDD. ∧ indicate that probabilities were measured by the corresponding software for each amino acid in the domain and a range is given. P(H) and P(C) represent the probability of a helix or coil at each amino acid (aa) in the domain. ∗ indicate that there were multiple hits in NCBI’s CDD, for which a range of E-values is presented. Click here for file

Additional file 2 **Table S1.** Class A scavenger receptor mRNA and protein sequence information. Novel sequences are indicated with bold font; those sequences marked as only predicted in GenBank and Ensembl databases are labeled with an asterisk. Proteins for which only partial sequence information is available is indicated with italics. Click here for file

Additional file 3 **Figure S1.** Phylogenetic trees of known and novel class A scavenger receptors indicate conservation of these receptors in a subset of vertebrate genomes. Phylogenies were created based on full-length cA-SR protein sequences. Novel sequences are indicated with bold font; those sequences marked as only predicted in GenBank and Ensembl databases are labeled with an asterisk. MARCO (a) was discovered in avian and mammalian genomes. SRAI (b) was found in organisms from *Xenopus* to mammals; no SRAI sequences were found in publicly available avian genomes. SCARA5 (c) was more phylogenetically diverse as 3 additional instances of this receptor were found in fish genomes. SCARA3 (d) was found in 2 Teleost fish genomes, *Danio rerio* and *Tetraodon nigroviridis*. SCARA4 (e) is the most phylogenetically widespread of the cA-SRs, present in 4 distinct fish species including the early bony fish of the superorder *Acanthopterygii*. Tree topologies were determined using both Bayesian and maximum likelihood methods and are supported by posterior probabilities and bootstrap values as indicated on node labels [BY/ML]. All phylogenies are midpoint rooted; scale bar indicates the number of substitutions per site. Click here for file

Additional file 4 **Table S3.** Exon structure for 3 representative species (human *Hs*, mouse *Mm*, and opossum *Md*) containing each of the 5 class A scavenger receptors. Exons are annotated as 5’UTR (untranslated region), CYTO (cytosolic), TM (transmembrane), AH (*α*-helical region), COL (collagenous region), SRCR (SRCR domain), LEC (Lectin domain), and 3’UTR (untranslated region). Accession numbers are from Ensembl Transcripts or mapping of mRNA to NCBI genomic sequence for XM_001370497. Numbers represent exon length in nucleotides, with values in brackets representing identified untranslated regions. Click here for file
