# Supplementary material for: The evolution of the class A scavenger receptors
Source: BMC Evol Biol. 2012 Nov 27;12:227. doi: 10.1186/1471-2148-12-227 (PMC3567963; doi:10.1186/1471-2148-12-227)
Supplement: Additional file 4 — Table S3. Exon structure for 3 representative species (human Hs, mouse Mm, and opossum Md) containing each of the 5 class A scavenger receptors. Exons are annotated as 5’UTR (untranslated region), CYTO (cytosolic), TM (transmembrane), AH (α-helical region), COL (collagenous region), SRCR (SRCR domain), LEC (Lectin domain), and 3’UTR (untranslated region). Accession numbers are from Ensembl Transcripts or mapping of mRNA to NCBI genomic sequence for XM_001370497. Numbers represent exon length in nucleotides, with values in brackets representing identified untranslated regions. [file 1471-2148-12-227-S4.pdf]

Supplementary Table 3: Exon structure for three representative species (human [*Hs*], mouse[*Mm*], and opossum[*Md*]) containing each of the five class A scavenger receptors. Exons are annotated as 5'UTR (untranslated region), CYTO (cytosolic), TM (transmembrane), AH (alpha helical region), COL (collagenous region), SRCR (SRCR domain), LEC (Lectin domain), and 3'UTR (untranslated region). Accession numbers are from Ensembl Transcripts or mapping of mRNA to NCBI genomic sequence for XM_001370497. Numbers represent exon length in nucleotides, with values in brackets representing identified untranslated regions.

GENE	ORG	*5'UTR*	*5'UTR /CYTO*	CYTO	*CYTO /TM*	*AH*	*AH*	*COL*	*COL*	*COL*	*SRCR*	*SRCR /3'UTR*	ACCESSION NUMBER
MSR1/ SRA	*Hs*	(118)	(107) 103		114	413	187	81	81	54	189	134 (788)	ENST00000262101
	Mm		(70) 10	106	114	410	187	81	90	54	189	134 (166)	ENSMUST00000026021
	Md			121	114	425	187	81	90	62			ENSMODG00000019061

GENE	ORG	*5'UTR*	*5'UTR /CYTO*	CYTO	*CYTO /TM*	*AH*	*COL*	*COL*	*COL*	*SRCR*	*SRCR /3'UTR*	ACCESSION NUMBER
SCARA5	*Hs*	(471)	(127) 112		129	675	81	99	57	198	137 (2189)	ENST00000354914
	Mm	(344)	(118) 112		129	675	81	99	48	195	137 (2105)	ENSMUST00000022610
	Md			112	129	675	81	99	57	198	137	ENSMODT00000020311, XM_001370497

GENE	ORG	*5'UTR /CYTO*	*CYTO /TM*	*AH*	*COL*	*COL*	*COL*	*COL*	*COL*	*COL*	*COL*	*COL*	*COL*	*COL*	*COL*	*COL*	*SRCR*	*SRCR /3'UTR*	ACCESSION NUMBER
MARCO	*Hs*	(232) 97	102	225	36	108	45	45	108	99	36	99	63	45	99	45	177	134 (274)	ENST00000327097
	Mm	(416) 108	102	219	36	99	45	45	108	99	36	99	63	45	99	45	177	131 (252)	ENSMUST00000027639
	Md		118	269	34	90	45	45	112	95	36	99	63	--	90	--	165	125	ENSMODT00000000530

GENE	ORG	*5'UTR /CYTO*	*CYTO*	*CYTO /TM*	*AH*	*AH*	*COL/3'UTR*	ACCESSION NUMBER
SCARA3	*Hs*	(27) 7	99	120	99	1044	452 (2121)	ENST00000301904
	Mm	(249) 7	99	120	99	1044	452 (1794)	ENSMUST00000042046
	Md		112	114	99	1044	452	ENSMODT00000020278

GENE	ORG	*5'UTR /CYTO*	*CYTO*	*CYTO /TM*	*AH*	*AH*	*COL*	*LEC*	*LEC*	*LEC*	*LEC/3'UTR*	ACCESSION NUMBER
SCARA4	*Hs*	(215) 7	51	123	99	1047	489	137	110	146	20 (704)	ENST00000400256
	Mm	(112) 7	51	123	99	1047	489	137	110	146	20 (1008)	ENSMUST00000040069
	Md		52	123	99	1047	489	137	110	163		ENSMODT00000027319
